# Chip-based quantum key distribution

**DOI:** 10.1038/ncomms13984

**Published:** 2017-02-09

**Authors:** P. Sibson, C. Erven, M. Godfrey, S. Miki, T. Yamashita, M. Fujiwara, M. Sasaki, H. Terai, M. G. Tanner, C. M. Natarajan, R. H. Hadfield, J. L. O'Brien, M. G. Thompson

**Affiliations:** 1 Centre for Quantum Photonics, H. H. Wills Physics Laboratory and Department of Electrical and Electronic Engineering, University of Bristol, Merchant Venturers Building, Woodland Road, Bristol BS8 1UB, UK; 2 National Institute of Information and Communications Technology (NICT), 588-2 Iwaoka, Kobe 651-2492, Japan; 3 National Institute of Information and Communications Technology (NICT), 4-2-1 Nukui-Kitamachi, Koganei, Tokyo 184-8795, Japan; 4 School of Engineering, University of Glasgow, Glasgow G12 8QQ, UK

## Abstract

Improvement in secure transmission of information is an urgent need for governments, corporations and individuals. Quantum key distribution (QKD) promises security based on the laws of physics and has rapidly grown from proof-of-concept to robust demonstrations and deployment of commercial systems. Despite these advances, QKD has not been widely adopted, and large-scale deployment will likely require chip-based devices for improved performance, miniaturization and enhanced functionality. Here we report low error rate, GHz clocked QKD operation of an indium phosphide transmitter chip and a silicon oxynitride receiver chip—monolithically integrated devices using components and manufacturing processes from the telecommunications industry. We use the reconfigurability of these devices to demonstrate three prominent QKD protocols—BB84, Coherent One Way and Differential Phase Shift—with performance comparable to state-of-the-art. These devices, when combined with integrated single photon detectors, pave the way for successfully integrating QKD into future telecommunications networks.

## Abstract (web summary)

Quantum key distribution has not been widely adopted in part due to technical hurdles preventing it being fully integrated in classical communication networks. Here the authors report quantum key distribution between two photonic chips manufactured with state-of-the-art telecoms industry processes.

Many of our conventional cryptographic schemes are based on the assumption of an adversary's computational power. In comparison, quantum key distribution (QKD) establishes cryptographic keys by transmitting single photons across a quantum channel, with its security based on the physical laws of quantum mechanics^1^,^2^. Over the last few decades, QKD has developed from simple demonstrations to robust implementations^3^,^4^,^5^,^6^, and is one of the first commercial quantum technologies^7^,^8^. Despite this maturity, QKD has seen limited adoption. Practical, large-scale deployment likely requires the use of integrated photonic devices providing enhanced functionality and miniaturization, in a platform amenable to mass-manufacture and easy integration with existing and emerging classical integrated telecommunications infrastructure.

While extreme levels of integration have been achieved in the microelectronics industry over the past decades, it is only recently that size, cost and power consumption considerations have demanded higher levels of integration in photonics. Fibre-to-the-home, data centre and 100 Gbps metro and long-haul network applications have driven the development of the indium phosphide (InP) platform to the point of full integration of laser sources, amplifiers, modulators and detectors^9^. Integrated photonics^10^ is thus poised to deliver major benefits to QKD technology and networks^11^,^12^,^13^ by allowing the miniaturization of components and circuits for hand-held and field deployable devices. It also provides highly robust manufacturing processes, which help reduce cost for personal devices. Finally, the complexity achievable with the integrated platform enables practical implementation of multi-protocol operation for flexibility, multiplexing for higher rates and additional monitoring and certification circuits to protect against side-channel attacks^1^ in a fibre network.

While there have been individual demonstrations of time-bin decoding^14^, miniaturization^15^ and reconfigurability^4^ in integrated devices, here we report QKD operation of complex devices that will allow the use of quantum secured communications in the applications described above. We use the InP platform to implement a monolithically integrated transmitter (Fig. 1a), consisting of a tunable laser, optical interferometers, electro-optic phase modulators (EOPM) and a p-i-n photodiode. We implement a receiver (Fig. 1b), consisting of a photonic circuit with thermo-optic phase shifters (TOPS) and reconfigurable delay line in the silicon oxynitride (SiO_*x*_N_*y*_) platform and off-chip single photon detectors. Both photonic systems are manufactured using state-of-the-art industrial fabrication processes and are designed for multi-protocol reconfigurable operation, here we demonstrate three important QKD protocols: BB84 (ref. 16), coherent one way (COW)^17^ and differential phase shift (DPS)^18^. We show performance of the photonic devices with clock rates up to 1.7 GHz, a quantum bit error rate (QBER) as low as 0.88% and estimated secret key rates up to 568 kbps, for an emulated 20 km fibre link. These devices are manufactured using the same fabrication processes as classical communications technology and microelectronics. Together with the development of integrated single photon detectors^19^,^20^,^21^, they point the way to seamless integration with existing and emerging classical communication systems.

## Results

### Integrated photonic devices

**Figure 1:**
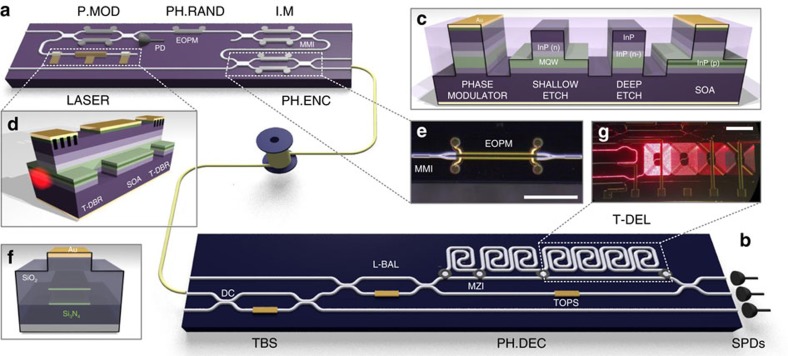
Integrated photonic devices for quantum key distribution. (**a**) A 2 × 6 mm^2^ integrated indium phosphide (InP) transmitter for GHz clock rate, reconfigurable, multi-protocol QKD. The circuit combines a continuous tunable laser diode (LASER), EOPMs, photodiode and interferometers formed by multi-mode interference (MMI) devices acting as 50:50 beamsplitters. This allows for pulse modulation (P.MOD), phase randomization (PH.RAND), intensity modulator (I.M) and phase encoding (PH.ENC). (**b**) A 2 × 32 mm^2^ silicon oxynitride (SiO_*x*_N_*y*_) photonic receiver circuit for reconfigurable, multi-protocol QKD that passively decodes the quantum information with off-chip single photon detectors (SPDs). MZIs are formed by directional couplers (DC), and configured with thermo-optic phase shifters (TOPS). This allows for a tunable beamsplitter, and a phase decoding (PH.DEC) circuit, which includes loss balancing (L-BAL) and a tunable delay (T-DEL). (**c**) The InP technology platform waveguide cross-section^9^ with the deep etch waveguide having 1 μm width and 4 μm etch depth. (**d**) Wavelength tunable continuous wave laser, formed from two tunable distributed Bragg reflectors (T-DBR) and a semiconductor optical amplifier (SOA) totalling 1.1 mm in length. (**e**) Microscopic image of EOPM in a MZI formed by two multi-mode interference devices acting as 50:50 beamsplitters. Scale bar, 500 μm. (**f**) The SiO_*x*_N_*y*_ Triplex waveguide cross-section, with metalisation for heating elements^22^ with a ∼2 μm waveguide width. (**g**) Microscopic image of the receiver delay lines. Scale bar, 1 mm.

Figure 1 shows a schematic of the chip-to-chip QKD system. For the transmitter device, the InP material system was chosen to meet the requirements of fast active electro-optics (with GHz operating speeds) and monolithic integration with the laser source. For the receiver device, the SiO_*x*_N_*y*_ material system was chosen to minimize photon loss from fibre-to-chip coupling and waveguide propagation loss, while maintaining a compact footprint. Both devices, along with fibre coupled single photon detectors, represent the full photonic QKD system.

### Transmitter

The InP-based transmitter chip was fabricated using an advanced active-passive integration technology^9^, where a multistep epitaxial growth process provides large flexibility in the waveguide structure (see Fig. 1c). The on-chip tunable laser (Fig. 1d) was formed from two distributed Bragg reflectors (DBR) and a semiconductor optical amplifier (SOA). When operated in continuous wave (CW) the laser source exhibited single mode behaviour with a coherence time >1.5 ns, a side-mode suppression ratio of >50 dB and an operating wavelength of 1,550 nm with ∼10 nm tuning range. Short electrical pulses applied to the reverse biased EOPM in the first Mach–Zehnder interferometer (MZI) enabled optical pulse generation with <150 ps duration and ∼30 dB extinction ratio. The exact timing between consecutive pulses could be accurately controlled by the driving electronics (see Supplementary Methods), and the on-chip photodiode was used to monitor the laser intensity and provide feedback to stabilize the laser current. The remaining EOPMs and MZIs were used to drive the different QKD protocols and to attenuate the laser pulses to the single photon level. Light was coupled out of the device using a lensed optical fibre, with the photon intensity levels calibrated at the output of this fibre.

### Receiver

The SiO_*x*_N_*y*_ receiver chip was fabricated using the TripleX technology platform^22^, where alternating layers of Si_3_N_4_ and SiO_2_ were deposited and etched to create a waveguide structure to guide light in a high index-contrast but low loss waveguides (∼0.5 dB/cm), and with low coupling loss between chip and fibre (∼2 dB), yielding a total loss ∼9 dB for BB84 configuration. While lower losses for integrated receivers using silica planar lightwave circuits have been reported^14^, our high index-contrast and small-footprint circuits allow for more complexity. This includes multi-protocol operation and multiple time-bin selection, but adds to the device loss.

Metal layers on top of the structure created TOPS for circuit reconfigurability. The first MZI acts as a tunable beamsplitter and taps off a portion of the incoming signal, which was routed to a single photon detector and used primarily for the COW protocol. The second MZI (L-BAL) acts to balance the losses in the asymmetric MZI (AMZI), which incorporates a digitally reconfigurable delay line, tunable from 0 to 2.1 ns in steps of 300 ps. This structure (PH.DEC) permits the interferometric measurement between the transmitter and receiver, and the TOPS within the AMZI was used to calibrate the phase relationship between the two arms of the interferometer. Light was coupled out of the device and into external fibre coupled superconducting nanowire single photon detectors mounted in a closed cycle refrigerator^23^ which had a system detection efficiency of ∼45% from the fibre input, a temporal jitter of ∼50 ps, average dark count rate of ∼500 cps, and a dead-time of ∼10 ns.

The highly reconfigurable nature of the transmitter and receiver devices allowed the implementation of a number of different QKD protocols. Here, we specifically investigated the three prominent protocols of BB84, COW and DPS. With these three protocols we demonstrate a number of key functionalities required for the transmission and reception of weak-coherent-based quantum key distribution. This includes high extinction ratio pulse modulation (periodic in DPS and non-periodic in COW), phase encoding (in DPS), phase randomization (for BB84), and intensity modulation (required for decoy states^24^). The commonalities of the receiver circuits include a combination of direct temporal measurements and phase interference, allowing for a reconfigurable generic design to accommodate the different transmission protocols. In each case, we have highlighted the specific security framework used for the analysis of the achieved secret key rates and compared to other key experiments in the literature.

### Protocols

**Figure 2:**
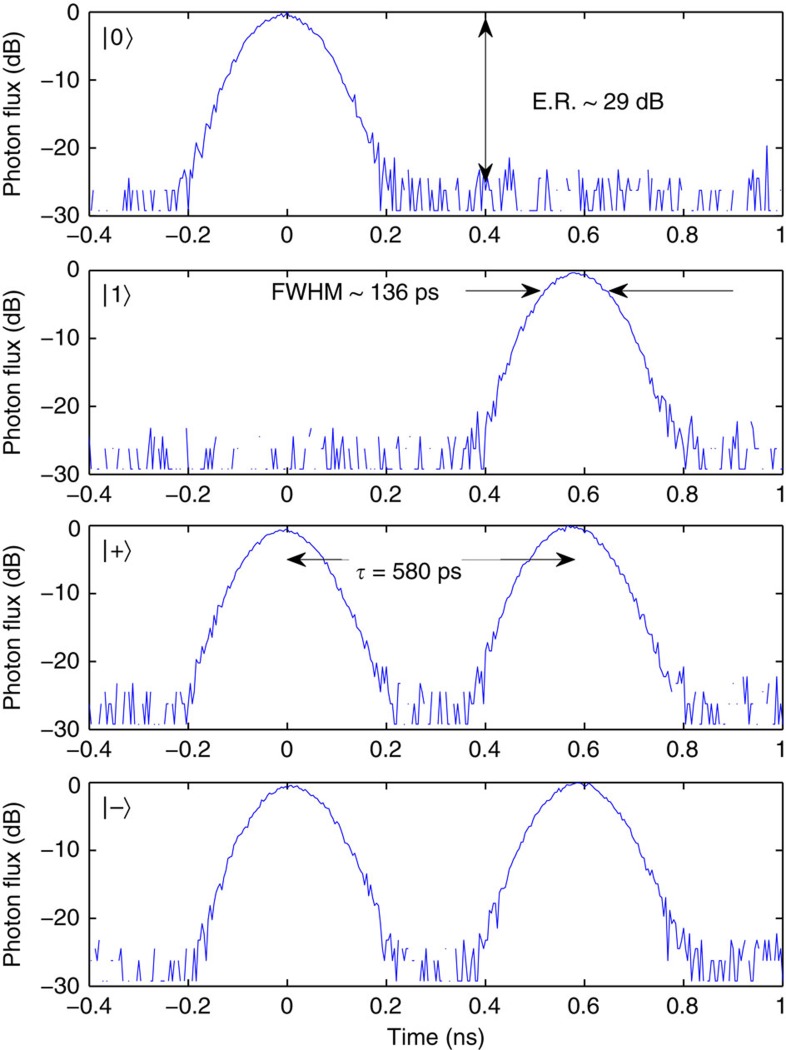
Transmitter output for the BB84 states. Single photon histogram measurements demonstrating the 136 ps full-width-half-maximum (FWHM) pulses with near 30 dB extinction. The two time-bins have a temporal separation of 580 ps, with a 0 or *π* relative phase difference for the |+> and |−> states, respectively.

The BB84 (ref. 16) QKD protocol was implemented using time-bin encoding, where |0> was encoded by a photon in the first time-bin and |1> was encoded by a photon in the second time-bin, while |+> was encoded by a photon in a superposition of the first and second time-bin with zero relative phase, and |−> was encoded by a photon in a superposition of being in the first and second time-bin with a *π* relative phase, as illustrated in Fig. 2. The BB84 protocol transmits one of two orthogonal states chosen at random, encoded in one of two randomly chosen non-orthogonal bases. We used the Z-basis {|0>, |1>} and the X-basis {|+>, |−>}.

The CW laser source was modulated (P.MOD) to select the time-bin choice, which was then phase randomised with a single electro-optic modulator (PH.RAND) before being attenuated and intensity modulated. The intensity of the {|+>, |−>} states was reduced by half, compared to the {|0>, |1>} states in order to maintain the same average photon number per state. The intensity modulator was also used to prepare one of three intensity levels at random to encode the ‘decoy' photon levels required for the security presented by Ma *et al*.^24^. The final MZI encoded the relative phase between successive time-bins to implement the |−> state.

Within the receiver chip, the digitally tunable delay line was reconfigured to match the 600 ps time interval between time-bins from the transmitter device. The phase decoding AMZI overlapped successive time-bins creating three possible time-slots within which to detect photons. Phase information interfered in the middle time-slot allowing measurements in the {|+>, |−>} basis, whereas time of arrival information in the first and third time-slots measured in the {|0>, |1>} basis.

The COW protocol^17^ transmits pulses in pairs, encoding |0> with the first bin and |1> with the second. Again the pulse modulated CW laser was used to generate pulses in these time-bins. While the key was generated unambiguously from the time of arrival of the single photon in a pair, security of the channel was determined by measuring the visibility from interfering successive photon pulses. A decoy state, with photon pulses in each time-bin (|0> and |1>), was included to increase the probability of occupied successive pulses, allowing a more accurate measurement of interference, and to detect photon-number-splitting attacks. Using the first MZI, the receiver routes a larger proportion of the input signal to single photon detectors for key generation, and a smaller proportion to the AMZI for visibility measurement.

Finally, the DPS protocol^18^ encodes information within the relative phase, 0 and *π*, of a train of photon pulses generated from the temporally modulated CW laser. The information was decoded unambiguously through the AMZI by interfering successive pulses, providing a QBER based on the number of incorrect counts at the wrong output of the phase decoding circuit. The security of the channel was determined by bounding the possible information an adversary could extract, that in turn would cause errors in the transmitted information.

### Rates

Each of the above three protocols were implemented on the chip-to-chip system, where the length of optical fibre link was emulated using a variable optical attenuator to induce channel loss. This was sufficient to demonstrate the dominant error mechanisms as the effects of dispersion are negligible for the broad ∼150 ps pulses used over these distances. A loss of 0.2 dB/km was assumed (standard within telecommunications fibres at 1,550 nm), although rates could be improved through use of low loss fibres^25^, and by optimizing the superconducting nanowire single photon detectors for ultra low dark counts^26^.

**Figure 3:**
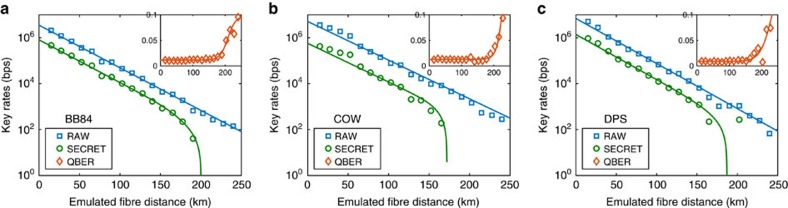
Experimental results. (**a**) BB84, (**b**) COW and (**c**) DPS showing the raw detection rate, estimated asymptotic secret key rate and relevant QBER. For BB84, the QBER is derived from the timing and phase errors, while for COW the QBER is derived from the timing error and security of the channel is estimated from phase coherence between successive pulses, and finally for DPS the QBER is estimated based on the error from the phase encoded information. State (or clock) rates of 560 MHz, 860 MHz and 1.76 GHz were used for BB84, COW and DPS, respectively.

Small fluctuations in the average count rates in Fig. 3 are due to slight variations in fibre-to-chip coupling efficiencies and would be reduced using standard v-groove fibre array packaging techniques, which should also provide facet coupling on the receiver of <1 dB. The emulated fibre distance in Fig. 3 represents the fibre length between the two systems, where each system includes the fibre-to-chip coupling loss of the packaged integrated device. This directly informs what can be expected once deployed in a real network.

The performance of our integrated devices for all three protocols is shown in Fig. 3, where the raw key rate, estimated asymptotic secret key rate, and QBER observed are plotted. For BB84, using an attenuation equal to 20 km of fibre we obtained an estimated secret key rate of 345 kbps using a clock rate of 560 MHz; using mean photon number pulses of 0.45, 0.1, and 5.0 × 10^−4^ for the signal and two decoy states chosen with probabilities of 0.8, 0.15, and 0.05 respectively; and observed an average QBER of 1.05%. The lower bound secret key rate for BB84 against general attacks was calculated using the raw and sifted key rates, and the measured QBER, using the security proof of Ma *et al*.^24^.

For COW, again using an attenuation equal to 20 km of fibre we obtained an estimated secret key rate of 311 kbps using a clock rate of 0.86 GHz with a QBER of 1.37% due to timing information and a QBER of 1.36% due to the interferometer and security of the channel. The secret key rate of COW was calculated using the sifted key rate and measured visibilities according to the security proof by Branciard *et al*.^27^ shown to be an upper bound for collective attacks. In order to use such security analysis, we additionally assume that the visibilities from any case of successively occupied pulses are all equal to the average value visibility measured across all cases. Finally, DPS at the same attenuation obtained an estimated secret key rate of 565 kbps using a clock rate of 1.72 GHz and measuring a QBER of 0.88%. The secret key rate of DPS was calculated by measuring the key errors and visibilities according to the upper bound security proof by Branciard *et al*.^27^ and is limited to collective attacks.

## Discussion

A summary of these results are presented in Table 1, where in all cases we show a performance comparable to the state-of-the-art in current fibre and bulk optical systems^1^. This work demonstrates the feasibility of using fully integrated devices within QKD systems, implementing three prominent protocols by utilizing the reconfigurability of the devices. The integrated photonic platform allowed us to demonstrate miniaturized devices exploiting robust, low-cost manufacturing processes, that allow flexibility in fibre network settings.

We have demonstrated key functionalities required for weak-coherent-based quantum key distribution, and these devices could be readily adapted to implement more protocols, such as the reference-frame independent^28^, SARG^29^ and B92 (ref. 30). Also the tunablity of the laser source enables flexibility in the wavelength of operation, this combined with the complexity achievable with the platform will be key to enabling high capacity wavelength division multiplexing schemes of the quantum channel in a practical implementation. Future demonstrations will require focus on the complete system for autonomous QKD operation, including the development of appropriate error reconciliation, privacy amplification and the use of finite-key analysis to qualify the security.

The increased complexity allowed by integrated photonics will facilitate the implementation of further monitoring and certification circuits, protecting against security flaws and side-channel attacks^1^ with minimal change in footprint and cost. For example the BB84 decoding used here allowed for a passive optical circuit, with the detection event constituting the random basis choice, thus removing the requirement for GHz rate active elements and quantum random number generators in the receiver. While this has been a common detection scheme in many different experiments, it can potentially open a security loophole, which can be mitigated with active basis selection^31^. Detector vulnerabilities^32^ could also be satisfied by operating the integrated devices for measurement-device independent QKD^33^.

Compatibility with current integrated photonic telecommunication hardware will ultimately allow seamless operation alongside classical communications transceivers, enabling hybrid classical and quantum communications devices. Moreover, the ability to scale up these integrated circuits to hundreds or even thousands of components^9^ opens the way to new and advanced integrated quantum communications technologies.

## Data availability

The data that support the findings of this study are available from the corresponding author upon reasonable request.

## Additional information

**How to cite this article:** Sibson, P. *et al*. Chip-based quantum key distribution. *Nat. Commun.* **8,** 13984 doi: 10.1038/ncomms13984 (2017).

**Publisher's note**: Springer Nature remains neutral with regard to jurisdictional claims in published maps and institutional affiliations.

## Supplementary Material

Supplementary Information Supplementary Figures, Supplementary Methods and Supplementary References.

## Figures and Tables

**Table 1:** Comparison of parameters and measured rates for three QKD protocols.

**Protocol**	**μ** **(per pulse)**	**State rate (GHz)**	**QBER time (%)**	**QBER phase (%)**	**Key rate (kbps)**	**Attack security**	**Key analysis**
BB84	0.45	0.56	1.17±0.18	0.92±0.11	345±15	General	Asymptotic
COW	0.28	0.86	1.37±0.15	1.36±0.16	311±50	Collective*	Asymptotic
DPS	0.28	1.72	N/A	0.88±0.10	565±89	Collective*	Asymptotic
BB84 (ref. 34)	0.42	∼1	Q_*X,Z*_ ∼{3.6, 4.3}		4,390	Collective	Finite
COW^25^	0.06	0.63	2.4	0.85	248	Collective*	Finite
DPS^35^	0.19	2.0	N/A	1.89	733	Individual	Asymptotic

Over an emulated fibre link of 20 km, assuming 0.2 dB/km, using a digital variable attenuator. Further example parameters for 20 km (4 dB) links for biased-basis BB84 (1.09 Mbps at 50 km)^34^, COW (12.7 kbps at 16.9 dB)^25^ and DPS (1.16 Mbps at 10 km)^35^ included for comparison. These values were either provided directly in the references or estimated/interpolated from the accessible data, and Q_*X*,*Z*_ refers to the two basis QBERs, which were not directly comparable to the time and phase QBERs demonstrated in this work.

^*^Indicates results based on the upper bound proofs of Branciard *et al*.^27^.
