# Supplementary material for: Chip-based quantum key distribution
Source: Nat Commun. 2017 Feb 9;8:13984. doi: 10.1038/ncomms13984 (PMC5309763; doi:10.1038/ncomms13984)
Supplement: Supplementary Information — Supplementary Figures, Supplementary Methods and Supplementary References. [file ncomms13984-s1.pdf]

Supplementary Information for Chip-based Quantum Key Distribution

P. Sibson,[1] C. Erven,[1] M. Godfrey,[1] S. Miki,[2] T. Yamashita,[2] M. Fujiwara,[3] M. Sasaki,[3] H. Terai,[2] M. G. Tanner,[4] C. M. Natarajan,[4] R. H. Hadfield,[4] J. L. O'Brien,[1] and M. G. Thompson[1]

[1]Centre for Quantum Photonics, H. H. Wills Physics Laboratory and Department of Electrical and Electronic Engineering, University of Bristol, Merchant Venturers Building, Woodland Road, Bristol BS8 1UB, UK.
[2]National Institute of Information and Communications Technology (NICT), 588-2 Iwaoka, Kobe 651-2492, Japan
[3]National Institute of Information and Communications Technology (NICT), 4-2-1 Nukui-Kitamachi, Koganei, Tokyo 184-8795, Japan
[4]School of Engineering, University of Glasgow, G12 8QQ, United Kingdom

SUPPLEMENTARY FIGURES

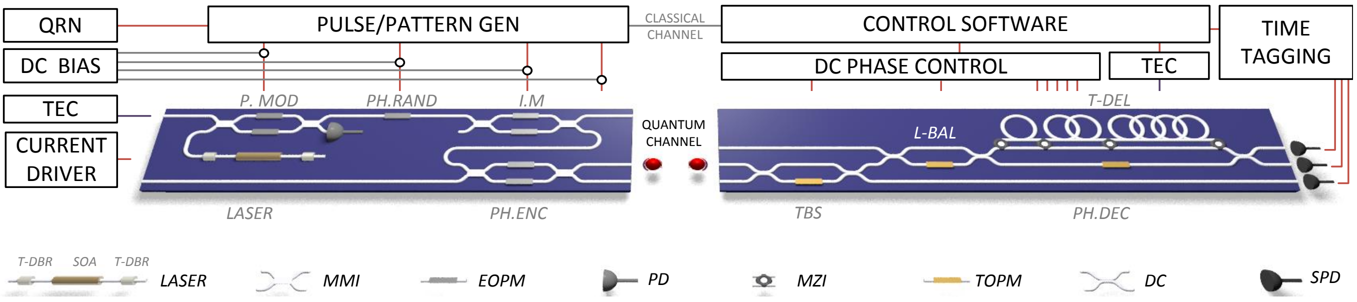

SUPPLEMENTARY FIGURE. 1: Schematic of the reconfigurable integrated InP transmitter "Alice" chip (left) which encodes quantum information on weak coherent laser light to be sent over optical fibre to a SiON receiver "Bob" chip (right). A temperature controller (TEC) and current driver stabilise the laser and control its intensity using feedback from an on-chip photodiode (PD). Electro-optic phase modulators (used to perform pulse modulation (P.MOD), phase randomisation (PH.RAND), intensity modulation (I.M), and finally phase encoding (PH.ENC)) are controlled through a combination of DC reverse biases (DC BIAS) and quantum random number (QRN) signals sent from a pattern generator through RF amplifiers. The receiver also requires a temperature controller (TEC) for phase stability and voltage sources (DC PHASE CONTROL) to control the thermo-optic phase modulators which determine the (passive) operation of the device. Off-chip superconducting nanowire single-photon detectors (SPD) detect the photons with time-tagging (TIME TAGGING) hardware, and control software synchronizes the experiment and post-processes the data.

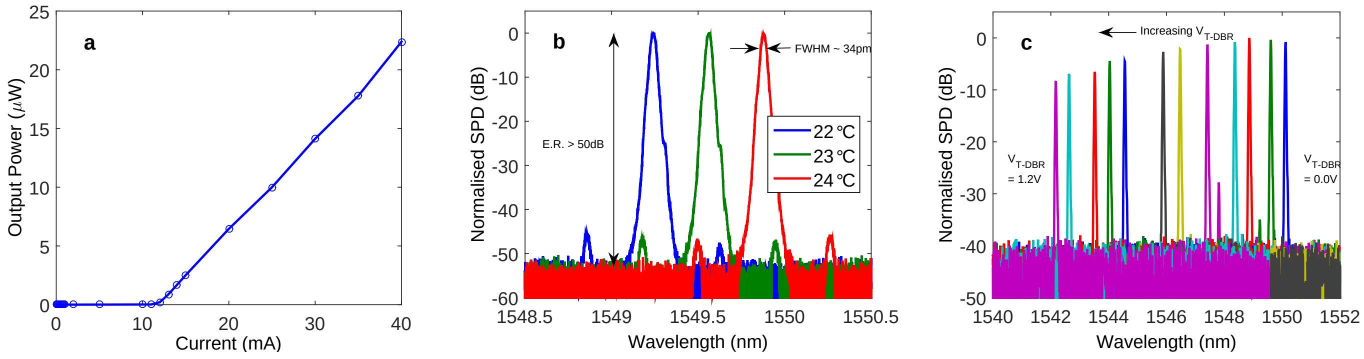

SUPPLEMENTARY FIGURE. 2: Characterisation of the on-chip laser showing (a) the output power versus driving current with a lasing threshold of ∼12 mA, (b) the tunability of the laser wavelength by adjusting the temperature of the device, and (c) the tunability of the laser wavelength by adjusting the voltages on the T-DBR.

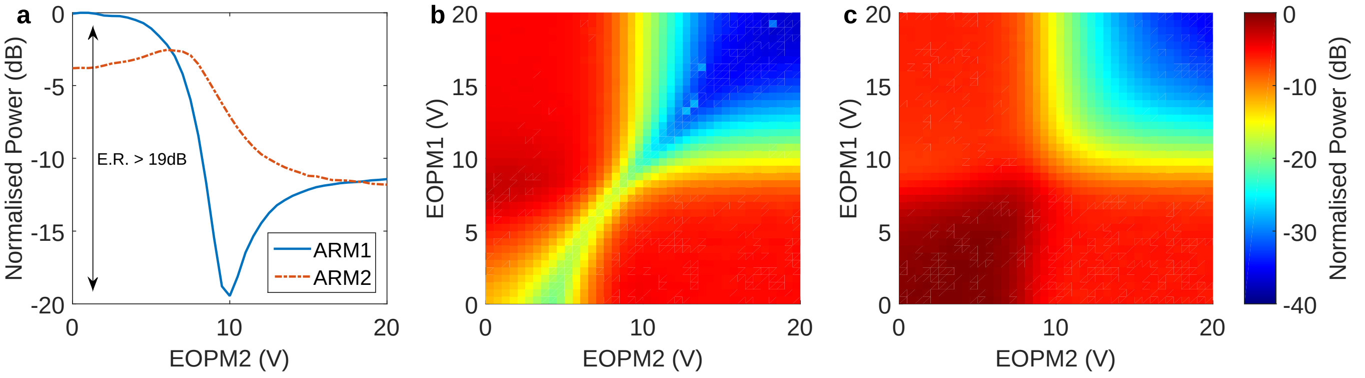

SUPPLEMENTARY FIGURE. 3: The intensity profiles measured during the DC characterisation of one of the MZIs. (a) Optical intensity output (at EOPM1 = 10 V) varied over EOPM2 voltage showing extinction ratio of over 19 dB for the output of the first output arm, and the heat map of intensity at the first output arm (b) and second output arm (c) as the two EOPMs in the Mach-Zehnder have their voltages varied.

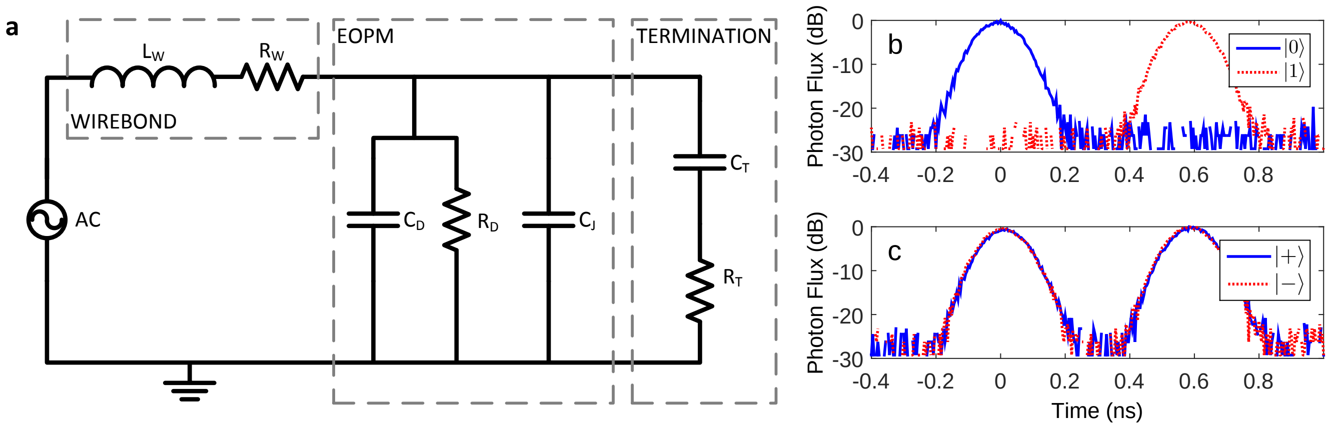

SUPPLEMENTARY FIGURE. 4: (a) Termination circuit for each EOPM, and (b) resulting pulse shape showing an extinction ratio of ~29 dB, FWHM of 134 ps, and separation of 580 ps between pulses.

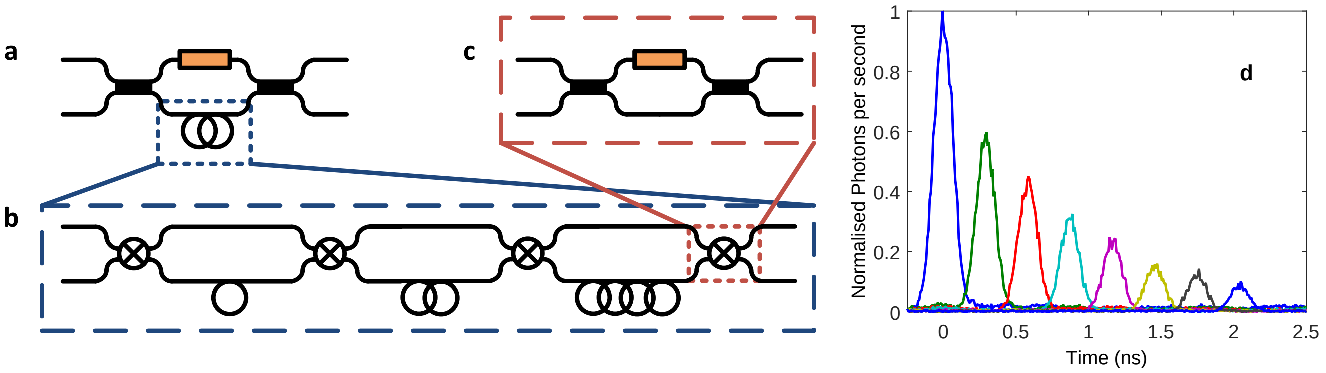

SUPPLEMENTARY FIGURE. 5: Schematic diagram of the (a) asymmetric Mach-Zehnder interferometer (AMZI), (b) digital delay lines, (c) MZI used to switch the signal into the different discrete delay line choices, and (d) the loss incurred for each of the possible digital delays.

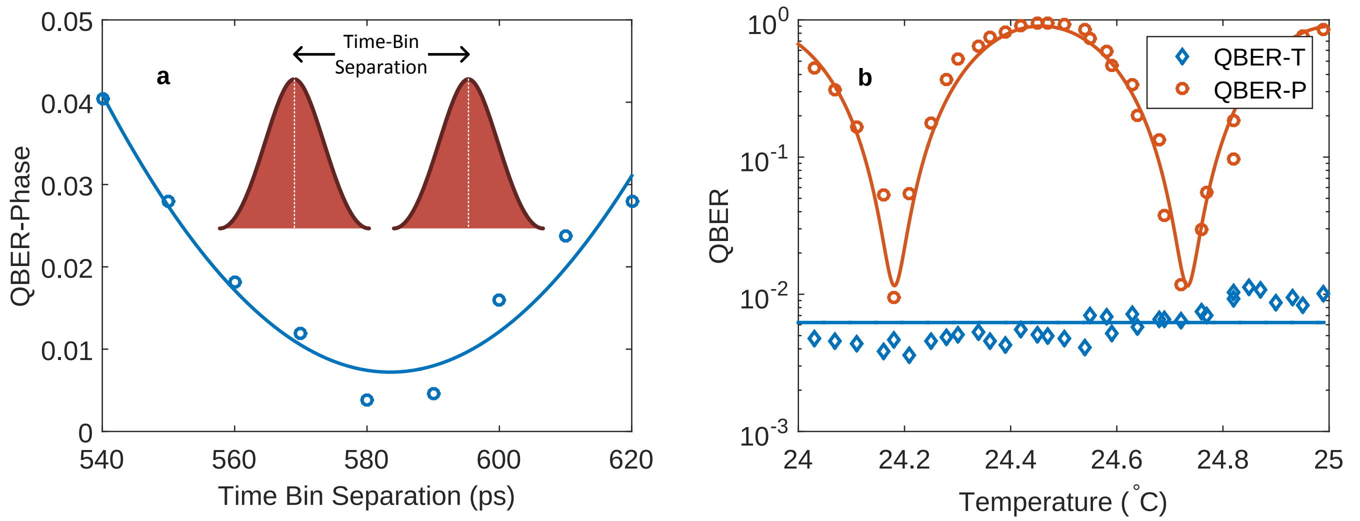

SUPPLEMENTARY FIGURE. 6: (a) Calibration of the optimum temporal delay, representing the time-bin separation, on Alice's transmitter which produces the lowest QBER when measuring qubits sent through Bob's receiver, and (b) the drift in the QBER due to the timing information (QBER-T) and phase information (QBER-P) as a function of the temperature of the receiver chip.

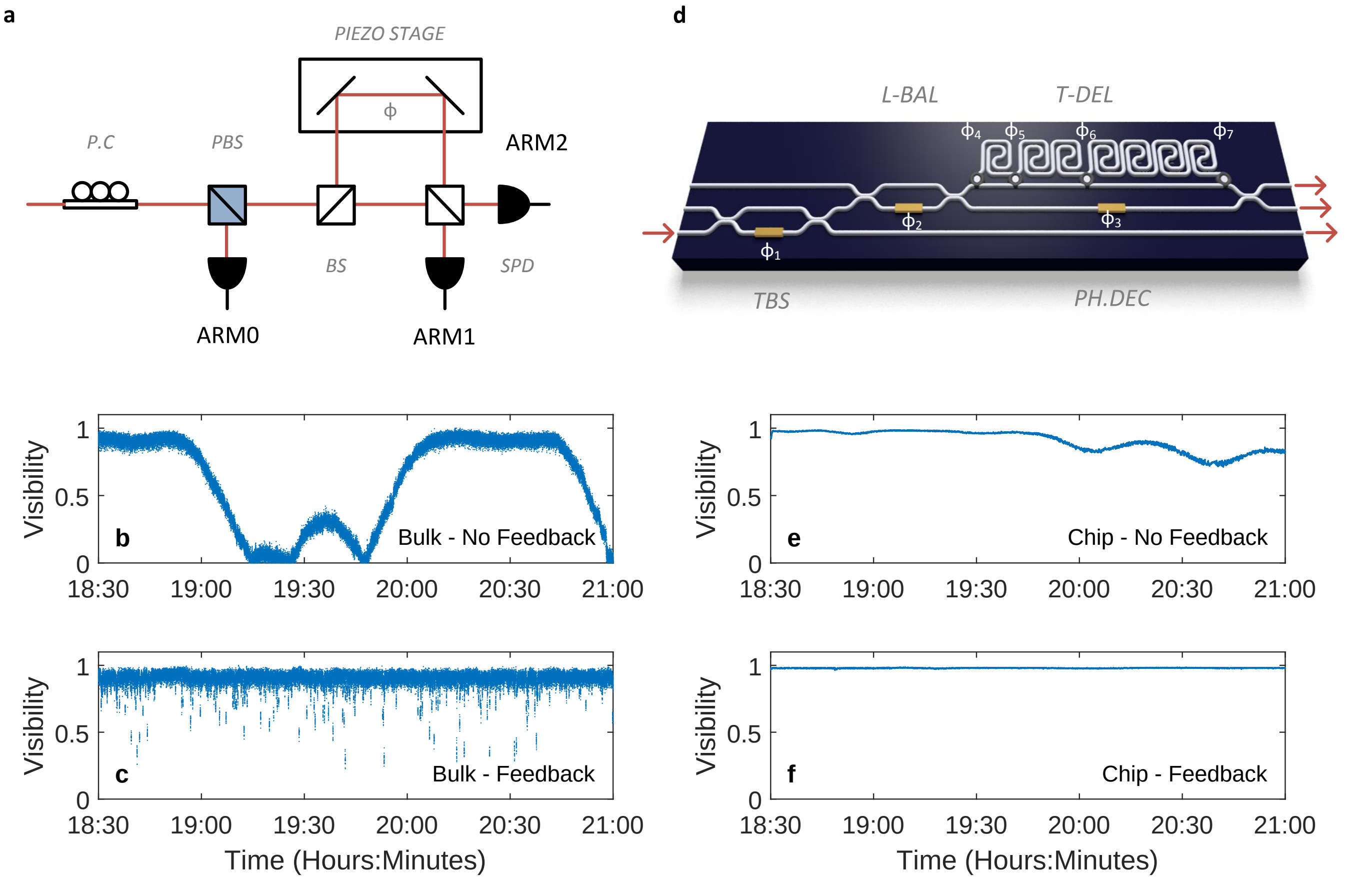

SUPPLEMENTARY FIGURE. 7: Interferometer Stability (a) Schematic of bulk optic interferometer (b) Visibility measure without feedback illustrating noise and drift over time (c) with gradient descent feedback on the piezo-stage, reducing the drift but still suffering from noise (d) Schematic of the integrated device interferometer (e) Visibility without feedback illustrating a slower level of drift (f) with feedback, showing a reduction in drift and noise.

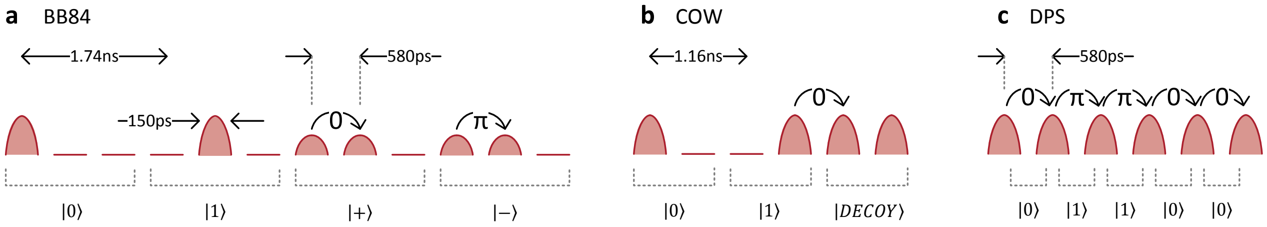

SUPPLEMENTARY FIGURE. 8: (a) The four states for the BB84 protocol, where $|0\rangle$ and $|1\rangle$ are encoded by photons in the first or second time-bin respectively, and $|+\rangle$ and $|-\rangle$ are encoded with a photon in a superposition of being in the first and second time-bin with a 0 and π phase shift respectively. (b) The three states for the COW protocol, where $|0\rangle$ and $|1\rangle$ are again encoded by photons in the first or second time-bin respectively and security is monitored by measuring the coherence across a pair of pulses from any combination of the $|DECOY\rangle$, $|0\rangle$, and $|1\rangle$ states. (c) The pulse-train used for the DPS protocol, where 0 and 1 are encoded as a phase of 0 or π respectively between pulses and security is monitored by measuring the coherence between pulses which shows up as errors in the measured bit values.

SUPPLEMENTARY METHODS

Our experimental configuration is shown in Supplementary Figure 1, with Alice on the left and Bob on the right. Alice is a fully integrated Indium Phosphide (InP) [1] QKD transmitter capable of being modulated at GHz rates at standard telecommunications wavelengths, while Bob is an integrated Silicon Oxynitride (SiO_xN_y, Triplex [2]) QKD receiver capable of passively measuring Alice's signals. The control hardware and software used to operate the integrated devices is also shown.

On the transmitter side, a temperature controller (TEC) stabilises the laser wavelength, while a current driver (CURRENT DRIVER) is used to control the intensity of the continuous wave laser using feedback from the photodiode (PD) via a transimpedance amplifier (TIA) when needed. Alice's electro-optic phase modulators (EOPM) are controlled through a combination of DC reverse biases (DC BIAS) and amplified RF voltage levels (not shown) from a pattern generator in order to perform pulse modulation (P.MOD), phase randomisation (PH.RAND), intensity modulation (I.M), and phase encoding (PH.ENC). For each qubit sent, the basis, bit, decoy intensities and phase randomisation are chosen using quantum random numbers (QRN) generated using Alice's chip prior to the key exchange.

The receiver also requires temperature control (TEC) for phase stability in the asymmetric Mach-Zender interferometer (AMZI) and voltage sources (DC PHASE CONTROL) to control the thermo-optic phase modulators (TOPM) determining the operation of the device. Off-chip super-conducting nanowire single-photon detectors (SNSPD) [3] are fibre coupled to the integrated devices and signals are time-tagged (TIME TAGGING) relative to a synchronisation signal sent from Alice. Control software is used to synchronise the system and compare the generated and measured signals allowing the extraction of the successfully distributed raw key, measurement of the QBER, and estimation of the secret key rate.

Integrated Transmitter Device

The integrated transmitter, shown in Supplementary Figure 1 (left), is a monolithically fabricated InP device comprised of a wavelength-tunable laser, electro-optic phase modulators (EOPM), and a photodiode. The EOPM are used in multiple interferometers to create intensity modulated and phase encoded weak-coherent signals for multiple-protocol quantum key distribution. The device was $2{\times}6$ mm^2 in size, compared to the equivalent discrete fibre based component systems measuring in area of $\sim$160$\times$15 mm^2 for a single packaged lithium niobate intensity modulator, and $\sim$25$\times$13 mm^2 for a single packaged laser diode, not including extra fibre lengths. Not only is there a decrease in size due to the monolithic fabrication, but there is also an ability to scale up in complexity.

Laser

The laser source is formed by a semiconductor optical amplifier (SOA) in an InP single-mode weak waveguide structure that is excited through carrier injection. The optical amplifier provides the gain medium and allows the laser to spontaneously emit over a wide spectral band. This is combined with two tunable distributed Bragg reflectors (T-DBR) on either side to create a Fabry-Perot cavity that resonates at specific wavelengths. The T-DBR are weakly index coupled gratings with uniform pitch, and are tunable via carrier injection.

The laser is driven by a laser diode current controller (Arroyo ComboSource 600) at ∼20 mA, but is adjusted to provide the desired phase relationship between successive pulses. Current versus optical power of the laser, including output coupling loss, is illustrated in Supplementary Figure 2 (a) which clearly shows a lasing threshold of ∼12 mA. The spectrum of the laser for different operating temperatures is shown in Supplementary Figure 2 (b) from which we see it has a ∼50 dB side-band suppression. The measured FWHM of the laser is 34 pm, but this is likely over-estimated due to the wavelength resolution of our optical spectrum analyser (Anritsu MS9740A OSA - with 0.03nm resolution). From the stable interference observed in our QKD experiments, we can instead state that our laser had a coherence time >1.5 ns. Temperature control and stabilisation is required to provide stable laser operation. The laser wavelength can also be tuned by carrier injection into the T-DBR, as shown in Supplementary Figure 2 (c). Using both effects we can easily tune the wavelength of the laser over a ∼10 nm range.

Photodiode

The photodiode is a ∼1GHz bandwidth, blocking (full absorption) waveguide-pin structure. This *p-i-n* photodiode structure is used to monitor the output from the laser signal to ensure stable laser intensity, by providing feedback for current input to the SOA.

Electro-Optic Phase Modulator

The electro-optic phase modulators (EOPM) operate through the Quantum Confined Stark Effect (QCSE) in a strong passive waveguide in which the core is a multi-quantum well (MQW) structure. By applying a reverse bias to this structure the QCSE causes a shift in the bandgap of the material incurring both phase and loss, dependent on the applied electric field. This effect, when oriented parallel to the InP crystal lattice, can have an additional linear electro-optic effect which increases efficiency. The QCSE is generally regarded as quadratic in nature, resulting in more nonlinear characteristics for phase shifters. It also has further wavelength dependence for both phase and loss, due to the proximity to the band-gap wavelength of the MQW core. By keeping the EOPM length less than 1 mm, the phase modulators allow up to 10 GHz in bandwidth, but also require careful placement on the die to allow good signal integrity to be maintained off-chip.

Supplementary Figure 3 (a) shows the intensity profiles from the DC characterisation of one of the Mach-Zehnder interferometers (MZI) on the transmitter chip, formed from two multi-mode interference devices (MMI) acting as 50:50 reflectivity beamsplitters and an EOPM in either arm. From this one can see an extinction ratio of near ∼19 dB can be achieved over low voltage changes. Supplementary Figure 3 (b) and (c) show the heat map of intensity at the two output arms for a full 2D sweep of the EOPMs. An important note is the EOPMs suffer from saturation which can limit the range of phase that can be applied if the devices are too short.

As illustrated in Figure 4, the EOPM is terminated with a parallel combination of a DC-blocking capacitor and AC-terminating 50 Ω resistor in series, allowing for high-speed RF operation. By concatenating two MZIs in series, one for timing intensity modulation (I.M) and one for phase encoding (PH.ENC), time bin encoded states can be prepared with an extinction ratio of ∼29 dB, FWHM of 134 ps, and pulse separation of 580 ps. This illustrates the high speed operation of our device and is given without compensating for detector jitter, time interval analysis, and trigger jitter.

Phase Encoding

To create $|+\rangle$ qubits, photon pulses are generated in the two temporal time bins with no phase change; however, the $|-\rangle$ state requires a π phase difference between the two pulses. Because of complications associated with driving

the EOPM's to V_π, we instead encoded the π phase shift by applying $\{0, \phi\}$ and $\{\phi, 0\}$ to the top and bottom EOPMs in the MZI (PH.ENC) during the first and second time bin respectively. The ratio of these two states is

$$\frac{1 - \exp(-\mathrm{i}\phi)}{\exp(-\mathrm{i}\phi) - 1} = -1 \tag{1}$$

which is equivalent to a π phase shift. We used this process to encode the π phase shift because we otherwise could not reach the required voltages or incurred too much loss or saturation in the EOPMs.

Reversing the combination of EOPM states also provides a π phase shift, but with a different global phase. This process was used to guide a calibration of the phase relationship and does not require the EOPMs to reach a full π range without loss, which is difficult to achieve with the QCSE driven EOPMs.

Phase Randomisation

Phase randomisation is required to match the security analysis in decoy state protocols [4, 5]. This is achieved by the single electro-optic phase modulator (PH.RAND). The phase for each qubit sent is randomly chosen from a set of 10 discrete phases between 0 and 2π which are themselves randomly chosen. This is sufficient to approach the asymptotic limit of perfect phase randomisation [6].

Quantum Random Number Generation

The quantum random numbers were generated offline, prior to key exchange, using Alice's transmitter chip as a quantum random number generator (QRNG) [7]. Her temporally modulated weak coherent source and an on-chip MZI acting as a beamsplitter were used to randomly split photons to one of two off-chip fibre-coupled superconducting nanowire single photon detectors, producing a random stream of 0's and 1's. To achieve a fair or appropriately biased coin, the values were fed through a Bernoulli factory [8] to provide coins with the required probability statistics. For example, an even output coin 0 followed by 1 is output as 0, and 1 followed by 0 is output as 1, and other pairs are discarded. These two outcomes have the same probability no matter the input bias of $\{p, (1-p)\}$ for choosing $\{0, 1\}$ and is most efficient when $p \sim 0.5$. Once generated, these quantum random numbers were used to set the basis, bit, decoy intensities, and phase randomisation for each qubit sent.

Integrated Receiver Circuit

The integrated receiver circuit, shown in Supplementary Figure 1 (right), is a monolitically fabricated SiO_xN_y device comprised of passive waveguides and thermo-optic phase shifters combined into interferometers to route signals and decode timing and phase information for multiple-protocol quantum key distribution. The photonic signals are detected by superconducting nanowire single photon detectors [3] to convert single photons into classical electronic signals. The entire chip is temperature controlled to maintain a constant phase relationship in the asymmetric interferometer structures used to decode phase information.

Thermo-Optic Phase Modulators

The thermo-optic phase modulators (TOPM) are a metal layer on top of the passive triplex waveguide structure causing a resistance. With a length of 1.5 mm the phase modulators have a resistance of ∼600 Ω and reach a 2π phase shift at ∼20 V. The thermo-optic coefficient is linear with power and therefore quadratic with voltage. We characterised them inside MZIs formed from two directional couplers acting as 50:50 reflectivity beamsplitters.

A minimum pitch between a heater and an adjacent waveguide of 250μm was estimated to achieve thermal crosstalk of ≤-17 dB. The effects of thermal crosstalk between neighbouring thermo-optic phase modulators was also negligible. To ensure this case the chips were temperature controlled with a Peltier device to allow heat sinking.

Loss

Propagation loss was measured to be around 0.5dB/cm, or 8.0dB/ns when considering a group index of around 1.7. The fibre to chip facet loss was between 1.7 - 5 dB loss depending on alignment and index matching gel with polarisation maintaining single mode fibre arrays.

Discrete Time Bins

The asymmetric MZI is used to decode the phase encoded information and allows for discrete time bins to be chosen as illustrated in Supplementary Figure 5 (a)-(b). Combinations of 1 bin, 2 bins, and 4 bins can be included in the delay by tuning MZI switches (Supplementary Figure 5 (c)), allowing for any delay between 0 and 2.1 ns in steps of 300 ps. Increasing the delay does bring with it increasing loss, as shown in Supplementary Figure 5 (d).

Calibration and Timing

To balance the loss inside the longer arm of the asymmetric MZI, there is a MZI (L-BAL) which routes a larger percentage of signal into the longer arm than the shorter arm. This percentage should allow for equal intensities in both arms at the recombination beamsplitter. To calibrate this tuning, the phase is swept and visibility is maximised.

To minimise the QBER due to the timing and phase errors, the temporal delay between the electronically defined time-bins on the transmitter chip was adjusted to match one of the fixed digital delays on the receiver chip. The optimum was found to be ∼580 ps, as shown in Supplementary Figure 6 (a). Supplementary Figure 6 (b) shows that sweeping the temperature of the chip does not affect the temporal information (QBER-T); however, the phase relationship between the two arms does change (QBER-P). The temperature corresponding to the minimum QBER-P was chosen.

Stability

To test the ability to encode the BB84 quantum states from the integrated transmitter device, a ($60\times60\times60$ cm^3) bulk optical receiver was constructed to allow for the decoding of the signals. Illustrated in Supplementary Figure 7 (a), the receiver contained a fibre-based polarisation controller before outputting to free space. Once in free space the path was incident on a polarisation beam splitter (PBS) that transmits horizontal and reflects vertical polarisation states. The combination of the polarisation controller and PBS allows a portion of the signals to be directed into a collection stage and towards a single photon detector (SPD) and the other portion in to an AMZI. The AMZI contains two 50:50 beam splitters (BS), and extra delay arm made from two mirrors. The mirrors were mounted on top of a translation stage for coarse temporal alignment of the delay which also had piezo control for fine control of the phase relationship between the two arms. To alter the phase by 2π would be 1.55 μm in free space, which was ∼4 V on the piezo driver. The two outputs of the AMZI were incident on fibre collection stages connected to the inputs of single photon detectors.

The entire system was enclosed in an insulating polystyrene box for temperature stability, and placed on an air-lifted optical bench to reduce vibrations. This still lead to noise and drift in the system as is evident in Supplementary Figure 7 (b) where we illustrate the visibility ($\frac{I_0-I_1}{I_0+I_1}$) over time. To compensate for this drift, a feedback loop was implemented that measured and optimised the visibility periodically using a gradient descent feedback scheme, using the piezo controller for fine phase control. This led to for a more consistent phase relationship (Supplementary Figure 7 (c)).

We further compare these results to the (2×32 mm^2) integrated receiver device illustrated in Supplementary Figure 7 (d). The chip was temperature controlled with a PID loop measuring a thermistor resistance and with a Peltier device to sink the heat generated from the thermo-optic phase modulators. Without any other feedback (Supplementary Figure 7 (e)) we see a less noisy visibility measurement and longer stability period. Supplementary Figure 7 (f) shows further improvement, by implementing a gradient descent feedback scheme with the thermo-optic phase shifter (ϕ_3) a stable visibility measurement was achievable, without the same level of fluctuations and noise seen in the bulk interferometer.

Superconducting Single Photon Detectors

This experiment utilised external fibre coupled superconducting nanowire single-photon detectors (SNSPD) [3] mounted in a closed cycle refrigerator which had a system detection efficiency of ∼45%, an average dark count rate of ∼500 cps, and dead times on the order of 10 ns. The operating voltage bias was chosen to optimise the signal to noise of the detectors, by limiting the dark counts compared to the single photon counts per second when signals are sent.

Further to the work demonstrated here, future systems will require the integration or the packaging of detectors to be included in the receiver system. Within superconducting detector technology there has been a number of examples of monolithically fabricated detector and photonic chips, with recent demonstrations in GaAs[9], and silicon substrates and waveguides [10–12].

Future quantum network scenarios may move towards centralised resources, such as the quantum access network [13] or "client-server" models [14]. This in turn may allow more resource expensive cryogenic cooling systems to be shared between many users, in a similar vain to the use of expensive ATMs and cheap credit cards.

More near term practical single photon detector for large scale deployment may use other detector technologies. Avalanche Photodiodes (APDs) utilises a highly reverse biased photodiode, in which an incident photon causes the breakdown of the diode junction, which in turn causes an avalanche of electrons, creating a detectable signal. This can have a downside of long recovery time (increasing the dead time), and afterpulses (false positive signals increasing the dark counts). To overcome some of these issues, the detectors are cooled, as well as gated (where the reverse bias is modulated, to only allow detection when photons are expected).

For telecommunication wavelength (1530nm to 1570 nm) more exotic materials are needed, such as InGaAs APDs, which have been recently shown to operate at 55% efficiency, with a 1 GHz gating, through the use of self-differencing detection [15]. These systems could introduce more noise (in terms of dark counts and afterpulses), more errors (in terms of timing jitter), and lower count rates (in terms of larger dead time) which in turn would limit the achievable distances and secure key rates demonstrated in this work.

Protocols

BB84

The BB84 quantum key distribution protocol [16] transmits 4 states, consisting of 2 orthogonal states in 2 non-orthogonal bases. In a time-bin encoding this can be achieved through $|0\rangle$ encoded by a photon in the first time-bin $|0\rangle_t|\alpha\rangle_{t-\tau}$, $|1\rangle$ encoded by a photon in the second time-bin $|\alpha\rangle_t|0\rangle_{t-\tau}$, $|+\rangle$ encoded by a photon in a superposition of being in the first and second time-bin with no relative phase change $|\frac{\alpha}{\sqrt{2}}\rangle_t|\frac{\alpha}{\sqrt{2}}\rangle_{t-\tau}$ or $|-\frac{\alpha}{\sqrt{2}}\rangle_t|-\frac{\alpha}{\sqrt{2}}\rangle_{t-\tau}$, and $|-\rangle$ encoded by a photon in a superposition of being in the first and second time-bin with a π relative phase change $|\frac{\alpha}{\sqrt{2}}\rangle_t|-\frac{\alpha}{\sqrt{2}}\rangle_{t-\tau}$ or $|-\frac{\alpha}{\sqrt{2}}\rangle_t|\frac{\alpha}{\sqrt{2}}\rangle_{t-\tau}$. The four states are illustrated in Supplementary Figure 8 (a).

The integrated transmitter modulates the continuous wave laser source, selecting the time-bin choice. The light is then phase randomised, attenuated and intensity modulated. Additionally, we attenuate the average photon number per pulse of the $\{|+\rangle, |-\rangle\}$ basis to half the intensity of the $\{|0\rangle, |1\rangle\}$ basis to maintain the same average photon number for each state. The final MZI encodes the relative phase between successive time-bins to implement a $|-\rangle$ state.

The four possible BB84 states enter the AMZI, which overlaps successive time-bins to allow phase information to interfere, creating three possible time-bins within which to detect photons. Measurement of a photon in the first or third time-bin in either detector constitutes a measurement in the $\{|0\rangle, |1\rangle\}$ basis with the first time-bin indicating a $|0\rangle$ and the third time-bin indicating a $|1\rangle$; whereas, measurement in the second time bin constitutes a measurement in the $\{|+\rangle, |-\rangle\}$ basis with the top detector indicating a $|+\rangle$ and the bottom detector indicating a $|-\rangle$. This design allows Bob to use a passive optical detection circuit, once the appropriate phases are set on the TOPM's, removing the need for high-speed quantum random numbers and an active basis selection in the receiver.

To increase data rates and range, the states are phase randomised and decoy intensity levels are used. This allows for the security rate to be calculated following the security proof of Ma *et al.* [5]

$$K_{\mathrm{BB84}} = \frac{1}{2} N_\mu \left\{ -Q_\mu f_{\mathrm{EC}} H_2(\epsilon_\mu) + Q_1^{\mathrm{L}} \left[1 - H_2(\epsilon_1^{\mathrm{U}}) \right] \right\}, \tag{2}$$

where N_μ is the number of signal intensity states sent per second, f_{EC} is the error correction efficiency (which we set

to $f_{\mathrm{EC}} = 1.2$ based on literature estimates), $H_2(\epsilon) = -\epsilon\log_2(\epsilon) - (1-\epsilon)\log_2(1-\epsilon)$ is the binary Shannon entropy, and Q_μ and ϵ_μ are the transmission probability and QBER respectively of a pulse with the signal intensity μ.

The remaining quantities Q_1^{L} and ϵ_1^{U} are estimated using the following formulas. The lower bound for the single photon transmittance, Q_1, which represents the probability that the signal state contains 1 photon and that the receiver detects just 1 photon, is calculated to be

$$Q_1 \geq Q_1^{\mathrm{L}} = \frac{\mu^2 \mathrm{e}^{-\mu}}{\mu\nu_1 - \mu\nu_2 - \nu_1^2 + \nu_2^2}\left[Q_{\nu_1}\mathrm{e}^{\nu_1} - Q_{\nu_2}\mathrm{e}^{\nu_2} - \frac{\nu_1^2 - \nu_2^2}{\mu^2}(Q_\mu \mathrm{e}^{\mu} - Y_0^{\mathrm{L}})\right], \tag{3}$$

where Q_i is the transmission probability of a pulse with the intensity $i \in \{\mu, \nu_1, \nu_2\}$, μ represents the signal state intensity, and (ν_1, ν_2) are the two decoy state intensities (weak and vacuum). Y_0^{L} is the lower bound for the count probability of an empty pulse, obtained from

$$Y_0 \geq Y_0^{\mathrm{L}} = \max\left\{\frac{\nu_1 Q_{\nu_2}\mathrm{e}^{\nu_2} - \nu_2 Q_{\nu_1}\mathrm{e}^{\nu_1}}{\nu_1 - \nu_2}, 0\right\} \tag{4}$$

Finally, the QBER for each intensity is represented as ϵ_i, and the upper bound for the single photon QBER, ϵ_1, is given by

$$\epsilon_1 \leq \epsilon_1^{\mathrm{U}} = \frac{\mu}{\nu_1 - \nu_2}\frac{\epsilon_{\nu_1}Q_{\nu_1}\mathrm{e}^{\nu_1} - \epsilon_{\nu_2}Q_{\nu_2}\mathrm{e}^{\nu_2}}{Q_1^{\mathrm{L}}\mathrm{e}^{\mu}}. \tag{5}$$

The effects of error correction and privacy amplification are considered without finite key analysis since the focus of this work is on the devices and the effects of finite key analysis depend sensitively on the amounts of key sent in a particular transmission.

COW

The distributed phase reference protocols require similar operations to BB84, including temporally defining states and implementing relative phases. In COW [17], the key information is encoded in the timing of the pulses while the security of the channel is monitored through the coherence of successive pulses. To implement COW, we transmit a train of light pulses, with an average photon number $\mu < 1$ in each pulse. At time t, for each pair of time-bins there are three possible states: $|0\rangle$ has a photon in the first time bin, represented as $|0\rangle_t|\alpha\rangle_{t-\tau}$; $|1\rangle$ has a photon in the second time bin, represented as $|\alpha\rangle_t|0\rangle_{t-\tau}$; and $|\mathrm{DECOY}\rangle$ consists of photons in both time bins $|\alpha\rangle_t|\alpha\rangle_{t-\tau}$. The three states are illustrated in Supplementary Figure 8 (b). In this representation τ is the repetition rate of the train of pulses.

To decode the information sent over the quantum channel a proportion of the signal is detected directly by an SNSPD while the rest of the signal is sent through an asymmetric MZI. The directly detected signals allow Bob to learn Alice's bit value through its time-of-arrival thus generating the key. The security of the channel is monitored through the coherence of successive photon pulses. This is done by setting the delay in the AMZI equal to the temporal period, τ, separating each pulse in the train of pulses so that successive pulses of light are overlapped and interfered, the visibility of which is monitored.

In this protocol, the phase between the pulses in the state $|\mathrm{DECOY}\rangle$ is not changed and therefore these should always constructively interfere and be output from the first arm when successive pulses of photons are sent. Further, Bob's device also measures the coherence across half a decoy pulse and either of the $|0\rangle$ or $|1\rangle$ states or even across the $|0\rangle$ and $|1\rangle$ states. The underlying mechanism of the visibility measurement does not differ between the $|\mathrm{DECOY}\rangle$ states or any other form of successively occupied time bins, and therefore all of these cases are used in estimating the coherence of the channel. This added benefit of having the coherence distributed both within and across signal separations means that Eve cannot count the number of photons in any finite number of pulses without introducing errors, thus prohibiting any photon number splitting (PNS) attacks [17, 18].

The derivation of a complete security analysis for COW is an on-going priority within the QKD academic community, including a proof of security against the photon number splitting attack and some collective attacks. For this work, we use the upper bound security analysis of Branciard *et al.* [18] and chose the mean photon number per pulse of 0.28 when intending to send a photon. We estimated the secure key rate using

$$K_{\mathrm{COW}} = R\left[1 - I_E^{\mathrm{COW}}(\mu) - f_{\mathrm{EC}}H_2(\epsilon)\right], \tag{6}$$

where R is the receiver raw key rate per second, and again $f_{\rm EC}$ is the error correction efficiency (which we set to $f_{\rm EC} = 1.2$ based on literature estimates), $H_2(\epsilon) = -\epsilon\log_2(\epsilon) - (1-\epsilon)\log_2(1-\epsilon)$ is the binary Shannon entropy. We calculate the remaining quantities as

$$I_E^{\rm COW}(\mu) = \epsilon + (1-\epsilon)H_2\left(\frac{1+F_V(\mu)}{2}\right), \tag{7}$$

where ϵ is the QBER, and $F_V(\mu)$ is given by

$$F_V(\mu) = (2V-1){\rm e}^{-\mu} - \xi\sqrt{1-{\rm e}^{-2\mu}}. \tag{8}$$

This yields a positive key rate when ${\rm e}^{-\mu} > \xi \equiv 2\sqrt{V(1-V)}$, where V is the measured visibility.

Further variants of the COW protocol have led to more general lower bound security proofs with more pessimistic rates that could be the focus of future work [19].

DPS

The Differential Phase Shift (DPS) QKD protocol [20] goes even further than COW and encodes the key information in the relative phases between successive pulses in a pulse train, where 0 phase difference encodes a $|0\rangle$, and a π phase difference encodes a $|1\rangle$, each with an average photon number of less than 1. The qubits are thus delocalised across more than one pulse, removing the PNS attack since splitting off a photon from a pulse does not reveal any key information to an eavesdropper. Alice creates a steady train of photon pulses (similar to repeating the $|+\rangle$ and$|-\rangle$ states from BB84) with her first MZI (P.MOD), attenuates the source through the intensity modulator (I.M), and, using quantum random numbers, randomly encodes either a 0 or π phase between each pulse representing the key bits 0 and 1 respectively, as shown in Supplementary Figure 8 (c).

The stream of data is sent directly to Bob where he uses his first MZI to direct all of the light to the top AMZI where successive pulses are overlapped to interfere constructively or destructively depending on the relative phase of successive photons. Referring to Supplementary Figure 1, his top detector fires when a 0 phase is measured, while his middle detector fires when a π phase is measured. Any attempt by an eavesdropper to listen in to the communication will necessarily disturb the coherence of these states and produce errors in the measurement results.

A mean photon number of 0.28 was chosen for each pulse [18], and the secure key rate was estimated by

$$K_{\rm DPS} = R\left[1 - I_E^{\rm DPS}(\mu) - f_{\rm EC}H_2(\epsilon)\right], \tag{9}$$

where $I_E^{\rm DPS}$ is calculated analogously to the COW protocol, but now the visibility and QBER are related by $\epsilon = \frac{1-V}{2}$. Unlike the COW protocol this result is not analytic, but the numerical calculations of Branciard *et al.* [18] show that its robustness under the same family of attacks is very similar to COW and is used as an estimate of the performance of distributed-phase-reference protocols in the presence of errors [17].

Further variants of the DPS protocol with block phase randomisation have led to more general lower bound security proofs with more pessimistic rates that could be the focus of future work [21].

Comparisons with other Results

The main text includes comparisons between this work and a sample of recent publications. We have included the result of Lucamarini *et al.* [22], which demonstrates an efficient implementation of BB84 through biased basis selection. The experimental finite key analysis illustrates security against collective attacks producing results of 1.09 Mbps at 50 km of standard (0.2 dB/km) fibre. We estimate the QBERs from Figure 3, and extrapolate the results back to 20 km distances (4 dB), and assumed a constant error rate over this range of distances. Later work has extended this analysis to prove security against more general attacks [23], and simulates that the percentage of secure key rate would not reduce dramatically, for example at 30 km the estimated key rate for collective attacks is 3.41 Mbps, and for general attacks this reduces to 3.12 Mbps (91.5%).

We further give examples of COW and DPS. The COW demonstration from Korzh *et al.* [24] implemented finite key analysis based on the upper bound security proof of Branciard *et al.* [18] against collective attacks for long range QKD over ultra-low loss fibres (∼0.16 dB/km). The data provided was 12.7 kbps secure rate at 16.9 dB (or 104 km), and extrapolated back to 20 km distances (4 dB). Providing the higher data rates this would likely reduce

the uncertainty of measurement and increase the proportion of secure rate in the finite case, and therefore the estimate provided of 248 kbps may be a slight under-estimate.

The DPS example from Wang *et al.* [25] provides asymptotic secure key rates under individual attack security. The data points provided include 1.16 Mbps at 10 km and 185 kbps at 50 km, from which we interpolate a result of 733 kbps for 20 km.

SUPPLEMENTARY REFERENCES

[1] M. Smit *et al.*, "An introduction to InP-based generic integration technology," *Semiconductor Science and Technology*, vol. 29, no. 8, p. 083001, 2014.

[2] A. Leinse, R. Heideman, E. Klein, R. Dekker, C. Roeloffzen, and D. Marpaung, "TriPleX; platform technology for photonic integration: Applications from UV through NIR to IR," in *Information Photonics (IP), 2011 ICO International Conference on*, pp. 1–2, May 2011.

[3] C. M. Natarajan, M. G. Tanner, and R. H. Hadfield, "Superconducting nanowire single-photon detectors: physics and applications," *Superconductor Science and Technology*, vol. 25, no. 6, p. 063001, 2012.

[4] H.-K. Lo and J. Preskill, "Security of quantum key distribution using weak coherent states with non-random phases," *Quantum Information and Computation*, vol. 7, no. 5&6, pp. 431–458, 2007.

[5] X. Ma, B. Qi, Y. Zhao, and H.-K. Lo, "Practical decoy state for quantum key distribution," *Phys. Rev. A*, vol. 72, p. 012326, July 2005.

[6] Z. Cao, Z. Zhang, H.-K. Lo, and X. Ma, "Discrete-phase-randomized coherent state source and its application in quantum key distribution," *New Journal of Physics*, vol. 17, p. 053014, May 2015.

[7] T. Jennewein, U. Achleitner, G. Weihs, H. Weinfurter, and A. Zeilinger, "A fast and compact quantum random number generator," *Review of Scientific Instruments*, vol. 71, pp. 1675–1680, Apr. 2000.

[8] A. A. Abbott and C. S. Calude, "Von Neumann normalisation of a quantum random number generator," *Computability*, vol. 1, no. 1, pp. 59–83, 2012.

[9] J. Sprengers, A. Gaggero, D. Sahin, S. Jahanmirinejad, G. Frucci, F. Mattioli, R. Leoni, J. Beetz, M. Lermer, M. Kamp, S. Höfling, R. Sanjines, and A. Fiore, "Waveguide superconducting single-photon detectors for integrated quantum photonic circuits," *Applied Physics Letters*, vol. 99, no. 18, p. 181110, 2011.

[10] S. Dorenbos, E. Reiger, U. Perinetti, V. Zwiller, T. Zijlstra, and T. Klapwijk, "Low noise superconducting single photon detectors on silicon," *Applied Physics Letters*, vol. 93, no. 13, p. 131101, 2008.

[11] F. Marsili, V. B. Verma, J. A. Stern, S. Harrington, A. E. Lita, T. Gerrits, I. Vayshenker, B. Baek, M. D. Shaw, R. P. Mirin, and S. W. Nam, "Detecting single infrared photons with 93% system efficiency," *Nature Photonics*, vol. 7, no. 3, pp. 210–214, 2013.

[12] C. Schuck, W. H. Pernice, O. Minaeva, M. Li, G. Gol'tsman, A. V. Sergienko, and H. X. Tang, "Matrix of integrated superconducting single-photon detectors with high timing resolution," *Applied Superconductivity, IEEE Transactions on*, vol. 23, no. 3, pp. 2201007–2201007, 2013.

[13] B. Fröhlich, J. F. Dynes, M. Lucamarini, A. W. Sharpe, Z. Yuan, and A. J. Shields, "A quantum access network," *Nature*, vol. 501, no. 7465, pp. 69–72, 2013.

[14] P. Zhang, K. Aungskunsiri, E. Martín-López, J. Wabnig, M. Lobino, R. W. Nock, J. Munns, D. Bonneau, P. Jiang, H. W. Li, A. Laing, J. G. Rarity, A. O. Niskanen, M. G. Thompson, and J. L. O'Brien, "Reference-Frame-Independent Quantum-Key-Distribution Server with a Telecom Tether for an On-Chip Client," *Phys. Rev. Lett.*, vol. 112, no. 13, p. 130501, 2014.

[15] L. C. Comandar, B. Fröhlich, J. F. Dynes, A. W. Sharpe, M. Lucamarini, Z. Yuan, R. V. Penty, and A. J. Shields, "Gigahertz-gated InGaAs/InP single-photon detector with detection efficiency exceeding 55% at 1550 nm," *Journal of Applied Physics*, vol. 117, no. 8, p. 083109, 2015.

[16] C. H. Bennett and G. Brassard in *Proceedings of the IEEE International Conference on Computers, Systems, and Signal Processing*, (New York), p. 175, 1984.

[17] V. Scarani, H. Bechmann-Pasquinucci, N. Cerf, M. Dušek, N. Lütkenhaus, and M. Peev, "The security of practical quantum key distribution," *Rev. Mod. Phys.*, vol. 81, pp. 1301–1350, Sept. 2009.

[18] C. Branciard, N. Gisin, and V. Scarani, "Upper bounds for the security of two distributed-phase reference protocols of quantum cryptography," *New J. Phys.*, vol. 10, no. 1, p. 013031, 2008.

[19] T. Moroder, M. Curty, C. C. W. Lim, L. P. Thinh, H. Zbinden, and N. Gisin, "Security of Distributed-Phase-Reference Quantum Key Distribution," *Physical Review Letters*, vol. 109, p. 260501, Dec. 2012.

[20] K. Inoue, E. Waks, and Y. Yamamoto, "Differential phase-shift quantum key distribution," in *Photonics Asia 2002*, pp. 32–39, International Society for Optics and Photonics, 2002.

[21] K. Tamaki, M. Koashi, and G. Kato, "Unconditional security of coherent-state-based differential phase shift quantum key distribution protocol with block-wise phase randomization," *arXiv preprint arXiv:1208.1995*, 2012.

[22] M. Lucamarini, K. Patel, J. Dynes, B. Fröhlich, A. Sharpe, A. Dixon, Z. Yuan, R. Penty, and A. Shields, "Efficient decoy-state quantum key distribution with quantified security," *Optics express*, vol. 21, no. 21, pp. 24550–24565, 2013.

[23] M. Lucamarini, J. F. Dynes, B. Fröhlich, Z. Yuan, and A. J. Shields, "Security bounds for efficient decoy-state quantum key distribution," *IEEE Journal of Selected Topics in Quantum Electronics*, vol. 21, no. 3, pp. 197–204, 2015.

[24] B. Korzh, C. C. W. Lim, R. Houlmann, N. Gisin, M. J. Li, D. Nolan, B. Sanguinetti, R. Thew, and H. Zbinden, “Provably secure and practical quantum key distribution over 307km of optical fibre,” *Nature Photonics*, vol. 9, pp. 163–168, Mar. 2015.

[25] S. Wang, W. Chen, J.-F. Guo, Z.-Q. Yin, H.-W. Li, Z. Zhou, G.-C. Guo, and Z.-F. Han, “2 GHz clock quantum key distribution over 260 km of standard telecom fiber,” *Optics letters*, vol. 37, no. 6, pp. 1008–1010, 2012.
